# Histamine Immunoreactive Elements in the Central and Peripheral Nervous Systems of the Snail, *Biomphalaria* spp., Intermediate Host for *Schistosoma mansoni*

**DOI:** 10.1371/journal.pone.0129800

**Published:** 2015-06-18

**Authors:** Mohamed R. Habib, Azza H. Mohamed, Gamalat Y. Osman, Ahmed T. Sharaf El-Din, Hanan S. Mossalem, Nadia Delgado, Grace Torres, Solymar Rolón-Martínez, Mark W. Miller, Roger P. Croll

**Affiliations:** 1 Medical Malacology Laboratory, Theodor Bilharz Research Institute, Giza, Egypt; 2 Department of Physiology and Biophysics, Dalhousie University, Halifax, Nova Scotia, Canada; 3 Zoology Department, Faculty of Science, Menoufia University,Shebin El-Kom, Egypt; 4 Institute of Neurobiology, University of Puerto Rico, Medical Sciences CampusSan Juan, Puerto Rico; 5 Department of Anatomy & Neurobiology, University of Puerto Rico, Medical Sciences Campus, San Juan, Puerto Rico; University of Calgary, CANADA

## Abstract

Histamine appears to be an important transmitter throughout the Animal Kingdom. Gastropods, in particular, have been used in numerous studies establishing potential roles for this biogenic amine in the nervous system and showing its involvement in the generation of diverse behaviours. And yet, the distribution of histamine has only previously been described in a small number of molluscan species. The present study examined the localization of histamine-like immunoreactivity in the central and peripheral nervous systems of pulmonate snails of the genus *Biomphalaria*. This investigation demonstrates immunoreactive cells throughout the buccal, cerebral, pedal, left parietal and visceral ganglia, indicative of diverse regulatory functions in *Biomphalaria*. Immunoreactivity was also present in statocyst hair cells, supporting a role for histamine in graviception. In the periphery, dense innervation by immunoreactive fibers was observed in the anterior foot, perioral zone, and other regions of the body wall. This study thus shows that histamine is an abundant transmitter in these snails and its distribution suggest involvement in numerous neural circuits. In addition to providing novel subjects for comparative studies of histaminegic neurons in gastropods, *Biomphalaria* is also the major intermediate host for the digenetic trematode parasite, which causes human schistosomiasis. The study therefore provides a foundation for understanding potential roles for histamine in interactions between the snail hosts and their trematode parasites.

## Introduction

Histamine (HA; imidazolethylamine) serves as a transmitter in the nervous systems of animals ranging from simple invertebrates to mammals [1][2][3][4]. Among the most intensively studied animals in this regard are the gastropod molluscs. In pioneering microchemical studies, high levels of HA and histidine decarboxylase activity were measured in specific individual neurons in the central nervous system (CNS) of the marine opisthobranch *Aplysia californica* [5][6][7]. The synthesis and metabolism of HA [8], and an active HA-transport mechanism [9] were also demonstrated in the CNS of this species where the biogenic amine was found to mediate a range of synaptic and modulatory actions [10][11][12][13]. More recently, immunohistochemical studies localized the distribution of HA-like immunoreactivity in neurons in all central ganglia and in sensory neurons associated with the statocysts in the central nervous system of *Aplysia* and a related marine opisthobranch, *Pleurobranchaea californica* [14][15][16]. In the pulmonate snails *Lymnaea stagnalis* and *Helix pomatia*, the distribution, uptake, release properties, and possible physiological actions of HA were studied by Hegedus et al [17], who found HA-like immunoreactive (HA-Lir) neuronal cell bodies in most ganglia and fibers in the neuropil of each ganglion, in all connectives, commissures, peripheral nerves, and in several peripheral tissues. Numerous sensory cells in the tentacles, lip, and statocysts of *Lymnaea* also displayed HA-like immunoreactivity [17][18][19], while administration of an HA antagonist disrupted behaviors involved in the orientation to gravity [19].

In the present study we investigate the distribution of HA in another genus of snail, *Biomphalaria*, which, like *Lymnaea* belongs to the gastropod clade Hygrophilia. *L*. *stagnatlis* and *Biomphalaria spp*. differ dramatically, however, having body plans that are mirror images to each other, with the former being a dextral species and the latter being sinistral species. Previous work on other closely related snails, such as *Helisoma*, and *Planorbis* has indicated that not only the ganglia but also individual cells can also be recognized as mirror image homologues in dextral and sinistral species [20][21][22][23]. The present study extends these comparisons to cells with a histaminergic phenotype.

Work on *Biomphalaria* also takes on added medical relevance since these snails serve as intermediate hosts for the human blood fluke *Schistosoma mansoni* that causes intestinal schistosomiasis [24][25][26], a disease that ranks second to malaria as the most prevalent parasitic disease in tropical countries [27][28][29]. Research suggests that HA and the other biogenic amines not only play important roles in the snails, but may also be crucial for survival of the parasite within the snail [30][31][32]. An increased understanding of the roles of these transmitters may therefore yield insights into both the behaviours and physiological functions of snail intermediate hosts, and could lead to targets for control of the parasite in regions where schistosomiasis is endemic. Previous studies have explored the possibility of a neural source of host-derived catecholamines and serotonin in the *S*. *mansoni*—*Biomphalaria* system [33][34][35]. The present study provides comparable localization of HA-containing neurons in *B*. *alexandrina* and *B*. *glabrata*, the principal intermediate hosts for *S*. *mansoni* in Africa and the Americas, respectively [36][37].

## Materials and Methods

### Collection and maintenance of *Biomphalaria*

*Biomphalaria alexandrina* were collected from irrigation canals in Giza Governorate, Egypt (approximate GPS coordinates N 29.6667°, E 31.2333°; http://www.fallingrain.com). The collection of snails from these public waterways is part of work that the Theodor Bilharz Research Institute conducts in coordination with the Ministry of Health to control snail populations in efforts to restrict the transmission of schistosomiasis. The collected snails were housed in the Medical Malacology Laboratory of the institute, in glass or plastic aquaria containing aerated tap water at room temperature (25 ± 1°C) on a 12:12 light/dark cycle. They were examined weekly for natural infections during a six week quarantine period and only uninfected, healthy snails were shipped to the Faculty of Medicine, Dalhousie University, Canada. Snails were fed daily with Romaine lettuce and tropical fish food (Nutrafin, R.C. Hagen, Inc., Montreal, QC, Canada). Protocols conducted on *B*. *alexandrina* were approved by the Animal Care Committee of Dalhousie University (Protocol #I13-06).

Specimens of *B*. *glabrata* were lab-reared in Puerto Rico. All snails were housed in glass or plastic aquaria at room temperature (21–25°C) and fed carrots or lettuce *ad lib*. Tanks contained distilled water with Instant Ocean (Kingman AZ, USA) added (1 g per 4L) to approximate pond water and crushed oyster shells or blackboard chalk as calcium supplements. Protocols conducted on *B*. *glabrata* were approved by the Institutional Animal Care and Use Committee (IACUC) of the University of Puerto Rico Medical Sciences Campus (Protocol #3220110).

### Central nervous system dissection

The snails used for these studies ranged from 8–12 mm in shell diameter and were considered to be sexually mature, as evidenced by their capacity to lay eggs. Before dissections, they were anesthetized by 10–20 min of incubation in cold 50 mM MgCl_2_. After removal from the shell, the head and foot regions were cut from the rest of the body and transferred to a Petri dish lined with Sylgard (Dow-Corning, Midland, MI, USA) for dissection in snail saline of the following composition in mmol L^-1^: NaCl 51.3, KCl 1.7, MgCl_2_ 1.5, CaCl_2_ 4.1, NaHCO_3_ 1.8, pH 7.8. The body walls were reflected and pinned laterally following an incision of integument along the dorsal midline. The reproductive and digestive organs were removed, and the ganglia were freed by severing their peripheral nerves. In order to obtain a flat mount of the isolated circumesophageal ring of ganglia, either the cerebral or pedal commissure was severed and the respective ganglia were reflected laterally.

Isolated central nervous systems were secured with minutien pins, treated with 0.5% protease (Type XIV; Sigma-Aldrich Chemical Co, Mississauga, ON, Canada or St. Louis MO; #P5147) in saline for 5–7 min, and rinsed in fresh saline prior to fixation. Brains were fixed in a freshly prepared solution containing 2% 1-ethyl-3(3-dimethylaminopropyl)-carbodiimide (Sigma-Aldrich; #E7750) and 0.4% N-hydroxysuccinimide (Sigma-Aldrich, #H7377) diluted in 0.1 M phosphate buffered saline (PBS; 50 mM Na_2_HPO_4_ and 140 mMNaCl, pH 7.4) for 3 hours at 4°C [38]. Tissues were transferred to vials containing 2% paraformaldehyde dissolved in PBS and the fixation was continued overnight at 4°C.

### Dissection and fixation protocol for the peripheral tissues

Processing of peripheral tissues followed the procedure of Wyeth and Croll [18] with some minor modifications: the lips, tentacles, foot, and mantle were dissected from *B*. *alexandrina* in saline with 0.125% 1-phenoxy-2-propanol (PP; Sigma-Aldrich; #484423) added as an anesthetic [39]. To improve the penetration of reagents, the peripheral tissues were incubated for 2 h in 0.25% collagenase (Sigma-Aldrich; #C9891) in PP-saline. Following several washes (4–6 x 5 min each) with fresh saline, the peripheral tissues were flattened between a glass coverslip and slide with modeling clay spacers at the corners [39], and then incubated at 4°C for 20–40 min. The flattened peripheral tissues were fixed for 1 h at 4°C under a coverslip with 250μl of 2% 1-ethyl-3(3-dimethyl-aminopropyl)-carbodiimide and 0.4% N-hydroxysuccinimide in PBS added slowly to one edge of the coverslip so that fixative flowed past the tissues. To complete fixation, peripheral tissues were transferred to 1 mL of the same fixative at 4°C for 2 h in a vial, followed by incubation in 2% paraformaldehyde in PBS overnight.

### Immunocytochemistry

Procedures modified from Hegedus et al. [17] and Wyeth and Croll [18] were used for wholemount immunocytochemistry of the central nervous system and peripheral tissues. Following fixation, the CNS and peripheral tissues were thoroughly washed in four changes of PBS and then bathed overnight at 4°C in a blocking solution of 1% bovine serum albumin (BSA; Sigma-Aldrich #A4503) in 0.25% Triton X-100 in PBS (PBS-T). The tissues were then incubated at 4°C for 7 days in the primary antibody (rabbit polyclonal anti-HA, Immunostar, Hudson, WI, USA; #22939) diluted in PBS-T (1:100). Tissues were then washed in PBS (3 x 1 h) and PBS-T (1 h) followed by incubation at 4°C for 5 days in donkey anti-rabbit AlexaFluor 555 or goat anti-rabbit AlexaFluor 488 antibodies (Invitrogen, Molecular Probes, OR, USA; #A31572, #A11008) diluted 1:100 in PBS-T containing 1% normal goat serum (Sigma-Aldrich #G9023). Following several additional washes in PBS, the tissues were mounted between glass coverslips in a 3:1 solution of glycerol to 0.1 M Tris buffer (pH 8.0) with 2% n-propyl gallate for imaging.

The specificity of the primary antibody was previously demonstrated in other molluscan species (*Aplysia californica* [14], *Macoma balthica* [40], *Lymnaea stagnalis* [17][19][18]). The specificity of the secondary antibodies used in this study was tested by omitting the incubation of tissues in anti-HA. This modification resulted in a complete lack of fluorescence in all specimens.

Laser scanning confocal image stacks of fluorescent immunohistochemical labeling were acquired using Zeiss Pascal, LSM 510 or 510 Meta microscopes. Series of optical sections at 0.5–1.5μm intervals were used to make maximum intensity projections using ImageJ (National Institutes of Health; http://imagej.nih.gov/ij/) or ZEN 2008 (Carl Zeiss, Inc. Germany). Plates were assembled and contrast adjustment of figures was implemented using Photoshop CS2 (Adobe Systems, San Jose, CA, USA) and Microsoft PowerPoint (v. 14.0, Microsoft Corp., Redmond, WA, USA). Schematic representations were created in Illustrator CS2 (Adobe Systems). Results reported in this study were observed in 25 specimens of each species.

### Connective backfills

Dissection protocols were performed as described above. Isolated ganglia from *B*. *glabrata* were positioned with minutien pins near a small petroleum jelly (Vaseline) enclosure (3–5 mm diameter) on the surface of a Sylgard-lined Petri dish. The cerebral-buccal connective was cut and its distal end was drawn into the Vaseline-lined pool. The saline was withdrawn from the pool and replaced with a saturated solution (1.4 mg / 50 μl dH_2_O) of biocytin (Sigma-Aldrich, St. Louis MO). The enclosure was sealed with Vaseline and the preparation was incubated overnight at room temperature to allow migration of the biocytin. The connective was then extracted from the pool and the ganglia were repinned and washed 3–5 times with saline. Following fixation in 4% paraformaldehyde (see [41]), tissues were transferred to microcentrifuge tubes, washed 5 times (30 min each) in 80 mM phosphate buffer containing 2% Triton X-100 and 0.1% sodium azide (PTA), and incubated in streptavidin conjugated to Alexa Fluor 546 (Molecular Probes, #S-11225) diluted 1:1,000 to 1:2,000 in PTA (24–48 h, room temperature). The preparations were assessed daily (1–5 days) until the quality of the backfill staining was determined to be sufficient for advancing to immunohistochemical processing to double label the preparations for HA-Lir.

## Results

HA-Lir neurons were widely dispersed throughout the central nervous systems of *B*. *alexandrina* and *B*. *glabrata* with the sizes, positions and numbers of cells appearing to be similar in the two species. Histaminergic cell bodies were observed in each of the central ganglia with the exception of the right pleural ganglion. Immunoreactivity was also abundant in the ganglionic neuropils, connectives and commissures. In addition to the neurons and axons that could be reliably identified in all or most specimens, the nervous system also contained some dimly labeled cells and axons that were variable from one specimen to another. Unless observed in several preparations, these faintly labeled elements are not described here. Finally, different body regions also contained numerous HA-Lir peripheral neurons, many of which appeared to be sensory in nature and immunoreactive axons were abundant in major peripheral nerves.

### Buccal ganglia

**Fig 1:**
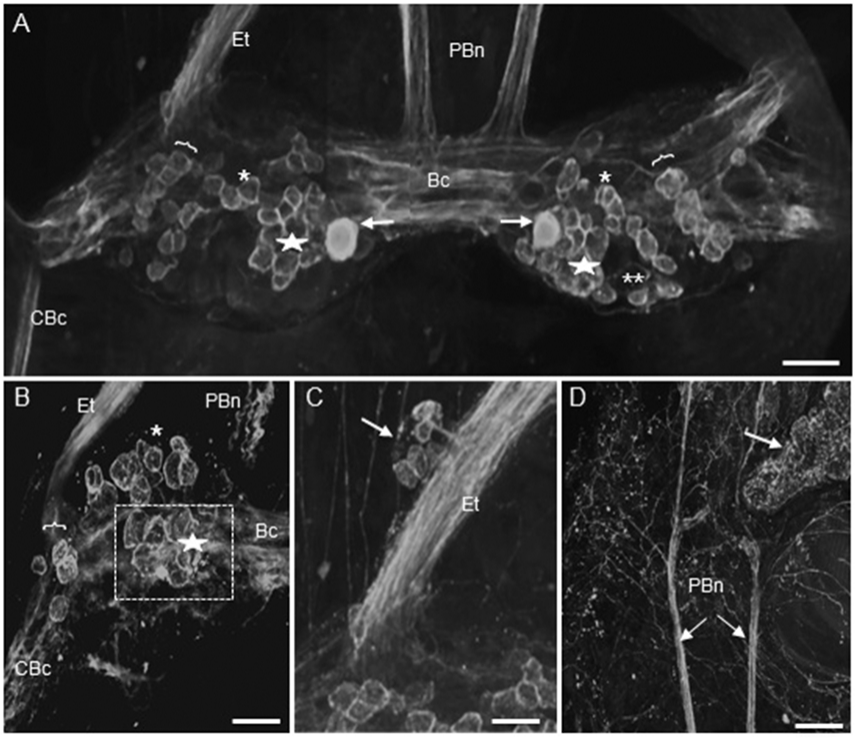
Histamine-like immunoreactivity in the buccal ganglia of *Biomphalaria*. **A:** Approximately 30 histamine-like immunoreactive neurons were present on the caudal surface of each buccal ganglion of *B*. *glabrata*. One population of neurons (stars) lay medially, and generally posterior to the buccal commissure. A single pair of larger (20–30 μm) more intensely labeled cells (arrows) flank the buccal commissure (Bc). A second population (single asterisks) formed an oblique band across each ganglion, while a third cluster (brackets) was located along the lateral margin. A few cells of a group located primarily on the ventral surface of the right ganglion are also indicated (double asterisks). The parabuccal nerves (PBn) and the esophageal trunks (Et) are rich in HA-immunoreactive fibers. Calibration bar = 50 μm. **B:** Cells comprising a medial cluster (star), central oblique band (single asterisk) and lateral population (bracket) can also be seen on the dorsal surface in a posteriorly rotated left buccal ganglion of *B*. *alexandrina*. Calibration = 50 μm. **C:** An encapsulated cluster (arrow) of immunoreactive neurons adhered to each esophageal trunk, near the ganglion. **D:** The parabuccal nerves project to the mouth region, where they branch to produce a rich innervation. Immunoreactive fibers also cover the salivary gland (arrow). Calibration bar = 100 μm.

Most of the HA-Lir neurons in the buccal ganglia in both *B*. *alexandrina* and *B*. *glabrata* were located on the dorsal surfaces. In both species three bilaterally symmetric populations of dorsal cells were reliably found although differences in rotation of the nearly spherical buccal ganglia during mounting sometimes distorted their boundaries and locations in different specimens (Fig 1A and 1B; also see summary diagram below). One cluster of about 15 immunoreactive neurons (15–20 μm diameter) was located posteromedially on the dorsal surface of each buccal ganglion near the buccal commissure (Fig 1A and 1B, stars). A particularly intensely stained cell was reliably observed within this population near the buccal commissure on each side (Fig 1A, arrows). A second population of 10–15 neurons formed a band extending from the posterolateral to anteromedial margins of the dorsal surface of each buccal ganglion (Fig 1A and 1B, asterisks). Neurons within this population appeared to be monopolar with some axons projecting toward the buccal commissure and others projecting into the underlying neuropil. We were unable, however, to follow any of these axons past their initial segments. A third population of about 10 neurons was located on the lateral margin wrapping between the dorsal and ventral surfaces of each buccal ganglion, near the origin of the cerebral-buccal connectives (Fig 1A–1C, brackets). An asymmetric cluster of 10–15 small neurons was located posteriorly on the ventral surface of only the right buccal ganglion (Fig 1A). In most of the preparations, 3–4 solitary weakly immunoreactive cells were also observed at various locations, e.g., near the origin of the cerebral-buccal connectives and on the ventral surface near the buccal commissure.

The neuropil, buccal commissure and all buccal nerves contained immunoreactive fibers. They were notably abundant in the cerebral-buccal connective, the esophageal trunk and the parabuccal nerves (Fig 1A–1D), which gave rise to a dense innervation of the buccal mass, including the salivary gland (Fig 1D). A capsule containing 5–8 HA-Lir neurons adhered to each esophageal trunk, approximately 100–200 μm distal to the ganglion (Fig 1C).

**Fig 2:**
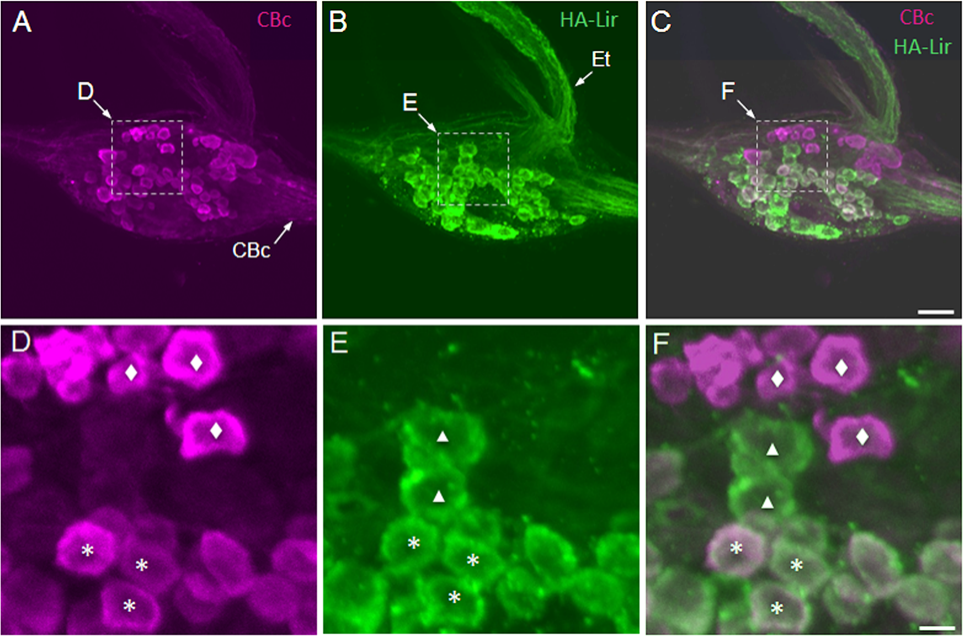
Double labeling of ipsilateral histamine-like immunoreactive neurons projecting toward the cerebral ganglion in *B*. *glabrata*. **A:** Biocytin backfill of the right cerebral-buccal connective (CBc) labeled numerous cells on the dorsal surface of the right buccal hemiganglion (magenta). Dashed box encloses area shown at higher magnification in panel D. **B:** Many of the histaminergic neurons on the dorsal surface of the ganglion (green) exhibited similar sizes and locations to those labeled by the backfill. Dashed box encloses area shown at higher magnification in panel E. **C:** An overlay of panels A and B demonstrates that approximately half of the dorsal buccal histaminergic neurons project to the CBc (double labeled neurons appear white). Dashed box encloses area shown at higher magnification in panel F. Calibration bar = 50 μm, applies to panels A-C. **D:** Higher magnification of central region of right dorsal buccal ganglion. Image contains neurons that were labeled only by the backfill (diamonds) and cells that also contained histamine-like immunoreactive material (asterisks). **E:** Same region of the ganglion contains neurons that were labeled only by the immunohistochemistry protocol (triangles) and others that were double-labeled (asterisks). **F:** Overlay of panels D and E confirms the presence of backfilled neurons (magenta, diamonds), histaminergic neurons (green, triangles), and double-labeled cells (white, asterisks). Symbols mark representative neurons, but not all cells in each class. Calibration bar = 10 μm applies to panels D-F.

**Fig 3:**
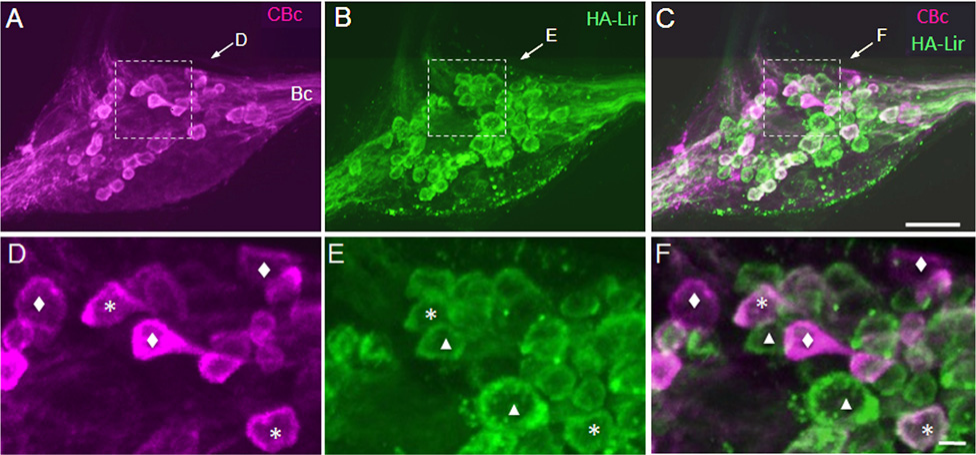
Double labeling of contralateral histamine-like immunoreactive neurons projecting toward the cerebral ganglionin *B*. *glabrata*. **A:** Biocytin backfill of the right CBc labeled fibers in the buccal commissure (Bc) and numerous cells on the dorsal surface of the left buccal ganglion (magenta). Dashed box encloses area shown at higher magnification in panel D. **B**: Many of the histaminergic neurons on the dorsal surface of the left ganglion (green) exhibited similar sizes and locations to those labeled by the backfill. Dashed box encloses area shown at higher magnification in panel E. **C:** Merging of panels A and B demonstrates that several histaminergic neurons project to the CBc (double labeled neurons appear white). Dashed box encloses area shown at higher magnification in panel F. Calibration bar = 50 μm, applies to panels A-C. **D:** Higher magnification of central region of the left dorsal buccal ganglion. Image contains neurons that were labeled only by the backfill (diamonds) and cells that also contained histamine immunoreactive material (asterisks). **E:** Same region of the ganglion contains neurons that were labeled only by the immunohistochemistry protocol (triangles) and others that were double-labeled (asterisks). **F:** Overlay of panels D and E confirms the presence of backfilled neurons (magenta, diamonds), histaminergic neurons (green, triangles), and double-labeled cells (white, asterisks). Symbols mark representative neurons, but not all cells in each class. Calibration bar = 10 μm applies to panels D-F.

The presence of numerous HA-Lir fibers in the cerebral–buccal connective prompted double-labeling experiments to test whether buccal histaminergic neurons could project to other central ganglia. Biocytin backfills of the cerebral–buccal connective resulted in labeling of 40–50 neurons on the dorsal surface of the ipsilateralbuccal ganglion (Fig 2A and 2D). When ganglia were subsequently processed for HA-Lir (Fig 2B and 2E), double-labeling was observed in approximately 20 neurons distributed across the central region of the ganglion (Fig 2C and 2F).A ventral histaminergic neuron near the buccal commissure was also double-labeled (not shown). When the contralateral buccal ganglion was examined, double labeling was present in 10–15 dorsal cells (Fig 3), consistent with the presence of HA-like immunoreactive neurons that project bilaterally to both cerebral-buccal connectives.

### Cerebral ganglia

**Fig 4:**
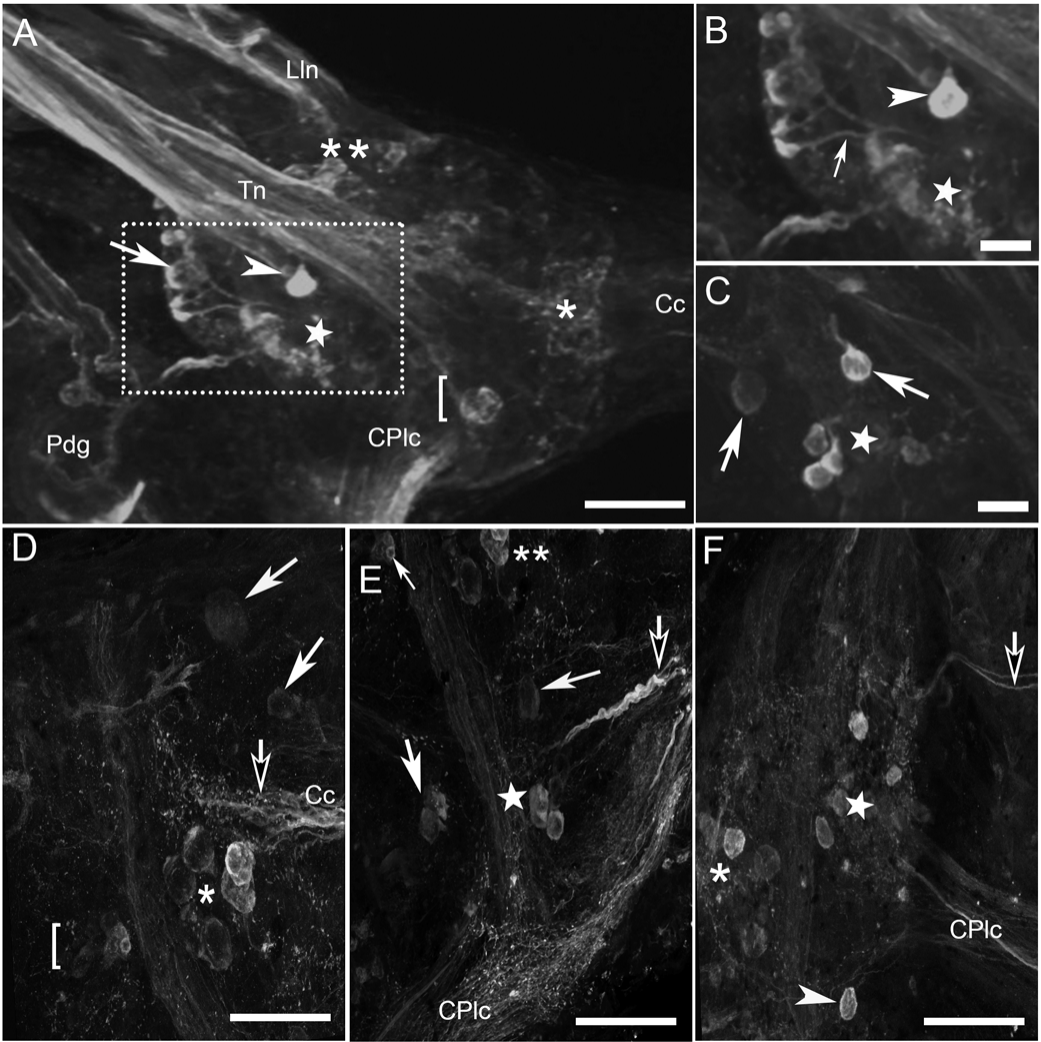
HA-like immunoreactivity in the cerebral ganglia. **A:** Dorsal view of the left cerebral ganglion of *B*. *glabrata*. The white arrow and double asterisks denote clusters along the lateral margin of the dorsal surface while the white arrowhead indicates an identified individual neuron at the base of the tentacular nerve (Tn). The star, single asterisk and bracket indicate ventral clusters that are not clearly in focus. **B:** Higher magnification view of area in part A indicated by the rectangle with the arrow showing an axon projecting from a cell in the lateral cluster. **C:** A deeper focus showing additional cells along the ventral surface in a similar region of another specimen of *B*. *glabrata*. **D:** Medial and, **E**: lateral regions of the right cerebral ganglia of *B*. *alexandrina*, as seen from a ventral vantage. The confocal z-stack spanned the thickness of the ganglion so that both dorsal and ventral cells are shown in the projection and many of the same cells and cell clusters indicated by stars, asterisks and double asterisks can be recognized here as described above for *B*. *glabrata*. One cluster of neurons (D, asterisk) is situated near the origin of the cerebral commissure (Cc) and another (E, star) near the cerebral-pleural connective (CPlc). The anterolateral cluster of neurons observed on the dorsal surface (A, double asterisk) continues to the anterior ventral surface (E, double asterisk) near the origin of the cerebral-buccal connective and lip nerves (out of view). Additional individual cells are located anterolaterally on the dorsal surface (E, large solid white arrows), and near the center of the ganglion on the ventral surface (D, large solid white arrows), and one solitary cell (E, small solid white arrow) at the base of the cerebral-buccal connective (diagonally above and out of view of this image). Numerous immunoreactive fibers can be observed in the cerebral commissures and connectives (large, solid white arrowheads). **F:** Ventral view of the left cerebral ganglia of *B*. *alexandrina*. Two clusters of neurons are observed, one (star) located at the origin of cerebral pleural connective (CPlc) and the other (asterisk) near the cerebral commissure (out of view to the left of this image). The subesophageal cerebral commissure contains a pair of fine immunoreactive filaments exiting the anterior ventral surface of the cerebral ganglia (contrasted black arrow). Scale bars = 50 μm, all panels.

The cerebral ganglia of *B*. *glabrata* and *B*. *alexandrina* contained numerous HA-Lir neurons in a range of sizes (Fig 4) and again homologous individual cells and clusters of cells could be recognized in the two species. Two prominent clusters of cells were located on the lateral margins of the dorsal surface of the each cerebral ganglion. The first cluster of small (10–15μm diameter) cells was located anterior to the origin of the tentacular nerve (Fig 4A, white double asterisks) while the second group of larger (15–20 μm) cells was located posterior to the nerve origin (Fig 4A and 4B, white arrows). A single intensely labeled neuron (15–20 μm) was observed overlying the base of the tentacular nerve located between these two clusters (Fig 4A and 4B, white arrowheads). Two additional dorsal neurons were reliably located as a separated pair of cells on the anterior dorsal margin of each cerebral ganglion (Fig 4D, large, solid white arrows).

On the ventral surface of each cerebral ganglion, one cluster of 5–6 neurons (~25 μm diameter) was located near the cerebral commissure (Fig 4A, 4D and 4F, single asterisks). A second cluster of 5–6 neurons was located more laterally on the ventral surface, near the cerebral-pleural connective (Fig 4A–4C, 4E and 4F, stars). This last cluster was noticeably asymmetric, with more cells extending closer to the origin of the cerebral-pleural connective in the left cerebral ganglion, compared to the right (see Fig 4E and 4F, stars). A final cluster of 4–5 cells was located near the base of the static nerve (Fig 4A and 4D, brackets). On the anterolateral margin, several cells from the previously described population of anterolateral dorsal cells continued onto the ventral surface of each cerebral ganglion (Fig 4E, double asterisks). Another pair of cells was found ventrally near the center of each ganglion (Fig 4E, large, solid white arrows). In addition, one large intensely stained cell (Fig 4C and 4E, small, solid white arrow) was situated ventrally at the base of the cerebral-buccal connective. Finally, at the origin of the superior lip nerve, a large oval HA immunoreactive bipolar cell appeared to project axons into the superior lip nerve and the sub-esophageal commissure (not shown but see summary diagram below).

Bundles of HA-like immunoreactive fibers were observed in the cerebral commissure between the two ganglia (Fig 4D and 4E, contrasted black arrows). Numerous immunoreactive fibers were also located in the lip and tentacular nerves (Fig 4A), and prominent HA-Lir axons were present in the cerebral-pleural connective (Fig 4A, 4E and 4F). The sub-esophageal cerebral commissure was noted in all preparations to contain a pair of fine immunoreactive filaments (2–3 μm diameter) exiting the anterior ventral surface of the cerebral ganglia (Fig 4F, contrasted black arrow).

### Pedal ganglia

**Fig 5:**
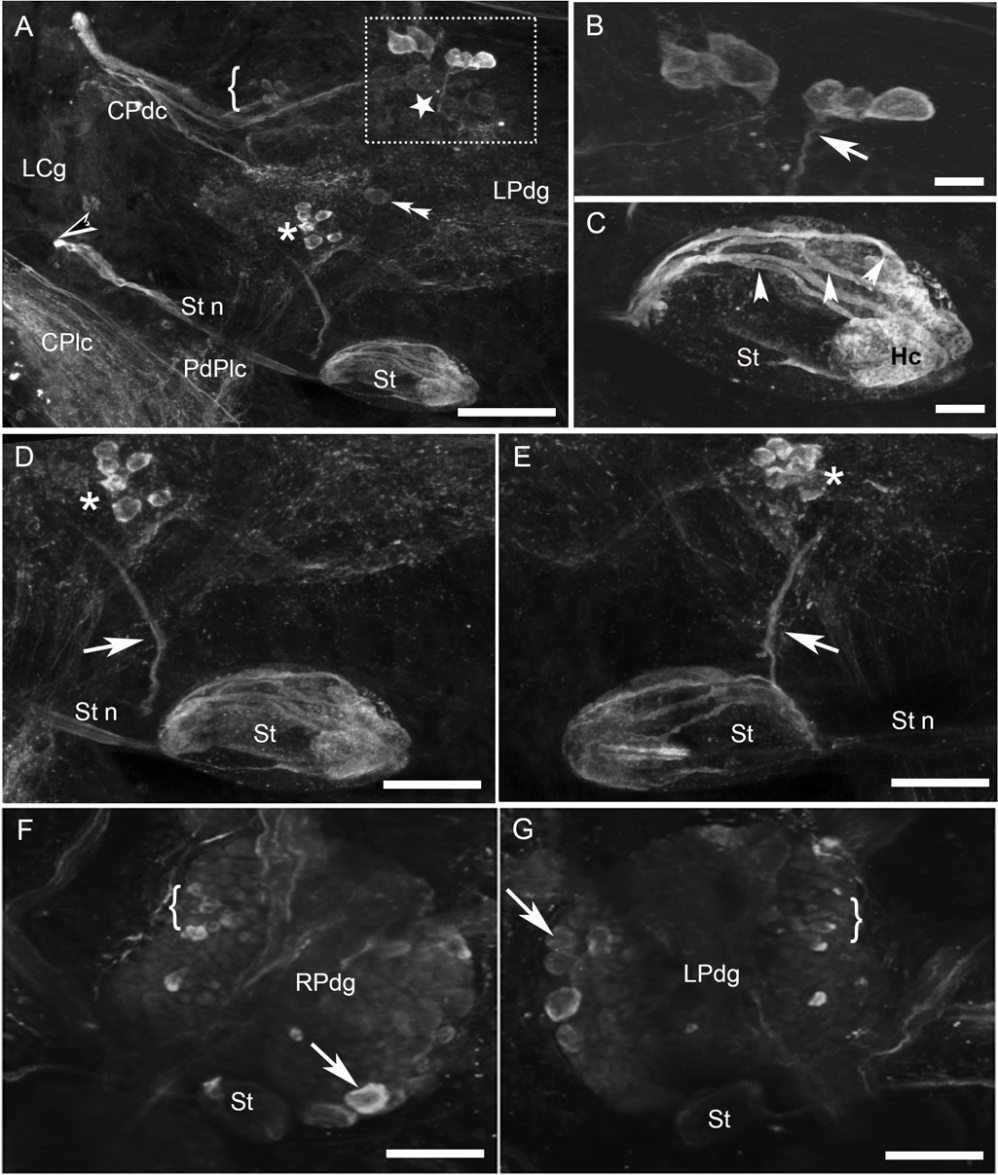
HA-Like immunoreactivity in the pedal ganglia of *Biomphalaria*. **A:** Two clusters of histaminergic neurons are located on the dorsal surface of the left pedal ganglion of *B*. *alexandrina*: one anteromedial cluster (rectangle) and a second cluster of smaller neurons (asterisk) near the origin of the pedal-pleural connective (PdPlc). The statocyst (St), located on the caudodorsal surface of the pedal ganglia, also contains immunoreactive somata and axons. The z-stack spanned the thickness of the ganglion and both dorsal and ventral cells are shown in the projection. A ventral cell lying near the center of the ganglion (double arrowheads) is also clearly seen although two anterior clusters of ventral neurons (indicated by a star and a bracket) are out of focus. Also, the continuous immunoreactive fiber system connecting the pedal and pleural ganglia through the pedal-pleural connective (PdPlc) can be seen, as well as the fibers connecting the cerebral ganglia to the pleural ganglia through their connective (CPlc). **B:** Closer view of the cluster of neurons on the anterior lateral edge showing the axonal projections directed toward the pedal commissure and the pedal nerves (out of view). **C:** Higher magnification of the statocyst (St) revealing the axons (solid white arrowheads) projecting out from the hair cells (Hc). **D, E:** The symmetry between the left pedal and right pedal ganglia, respectively, is illustrated by similar clusters of cells situated in each ganglion (asterisks), the same shape and position of statocysts (St) projecting the static nerve (Stn), and a thick bundle of fibers is running beneath the statocyst toward the pleural ganglia (arrow). **F, G:** Ventral views of the right and left pedal ganglia, respectively, of *B*. *glabrata*. The focal planes are toward the middle of the ganglia and therefore the outline of statocyst (St) can be seen, but the more dorsally positioned HA-Lir neurons within it are not visible, nor are dorsal cells in the ganglion. Cells of one of the ventral clusters visible in *B*. *alexandrina* (bracket) are indicated. Large (20–40 μm) neurons on the posterior and medial margins of the ganglia exhibit variable immunoreactivity. Calibration bars: A, D-G = 50 μm, B, C = 25 μm.

One cluster of relatively large (20–25 μm diameter), intensely stained neurons was located anteriomedially on the dorsal surface of each pedal ganglion (Fig 5A, rectangle). A higher magnification of this cluster (Fig 5B) showed the presence of monopolar cells with some axons (not shown) projecting toward the pedal nerves while others projected toward the pedal commissure in a tight bundle. A second cluster of dimly stained neurons was located on the ventral surface beneath the first cluster (Fig 5A, bracket). A third cluster of 6–8 intensely stained neurons about 10 μm in size was located more caudally on the dorsal surface, near the origin of the pedal-pleural connectives (Fig 5A, 5D and 5E, asterisks). A fourth cluster of small neurons (10–15 μm diameter) was located laterally on the ventral surface of both pedal ganglia near the origin of the cerebral-pedal connectives (Fig 5A, 5F and 5G, bracket). A single cell was located centrally on the ventral surface of each pedal ganglion (Fig 5A, double arrowheads). The axon of this cell projected toward the cerebral-pleural connective. In most of the preparation, three large, oval, weakly stained neurons were detected along the medial margin of the ventral surface of each ganglion posterior to the pedal commissure (Fig 5F and 5G).

As observed in the cerebral and buccal ganglia, HA-Lir fibers were present in the pedal commissure and in the connectives between the pedal ganglion and both the cerebral and pleural ganglia (Fig 5). Immunoreactive fibers were also observed in all of the major pedal nerves. A prominent, thick HA-Lir measuring 5 μm diameter also projected from the pedal ganglion toward the pleural ganglia, beneath each statocyst (Fig 5D and 5E, solid white arrows).

### Statocysts

The statocysts are attached to the caudodorsal surface of the pedal ganglia and embedded in connective tissue (Fig 5A and 5C–5G). While the majority of hair cells located in the medial half of the statocysts reliably displayed strong immunolabelling, the cells located laterally were not discernible in some preparations or exhibited only weak labeling in others (Fig 5C–5E, St). Immunoreactive axons (Fig 5C, solid white arrowheads) in the static nerves originated from the hair cells (Fig 5C, Hc) and projected to the isipilateral cerebral ganglion where they arborized in a circumscribed region of the neuropil (Fig 5A, contrasted black arrowhead).

### Pleural, parietal, and visceral ganglia

In the left pleural ganglion, a single large cell was consistently located near the center of the ventral surface, while no cells were detected in the right pleural ganglion in either species (data not shown but see summary diagram below).

**Fig 6:**
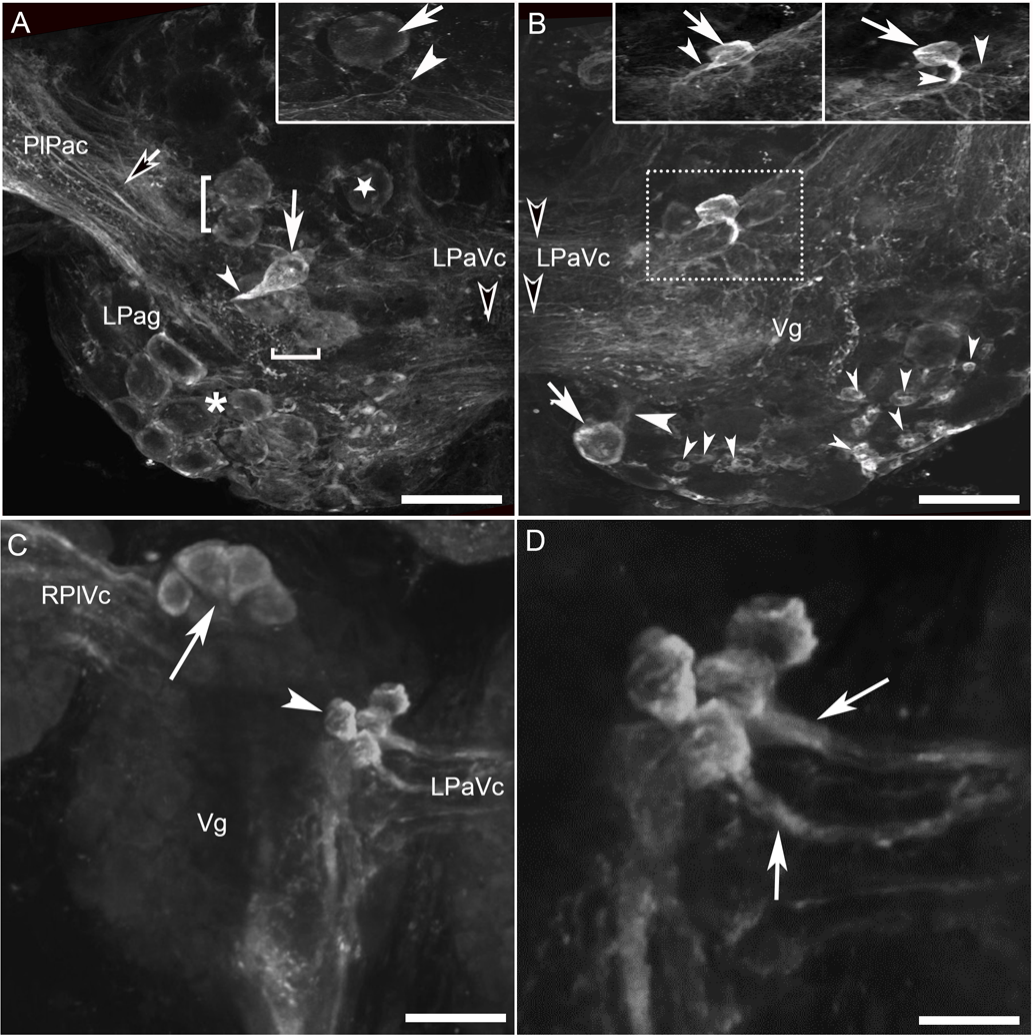
HA-Like immunoreactivity in the left parietal and visceral ganglia of *Biomphalaria alexandrina*. **A:** Left parietal ganglion contains a single large, intensely immunoreactive neuron (large solid white arrow) on the dorsal surface near the center of the ganglion with a thick axon projecting into the underlying neuropil (small solid white arrowhead). This cell near two pairs of lightly stained, large neurons (brackets). A large dim oval cell (inside rectangle) is located anterior to the parietal-visceral connective (LPaVc) projecting an axon toward the visceral ganglion (insert). A cluster of large, lightly stained cells (asterisk) can be seen on the posterior and lateral edge of the dorsal surface. Numerous fibers run in the parietal-pleural connective (PaPlc) including some prominent, thick axons (contrasted black arrow). **B:** Visceral ganglion. A prominent intense immunoreactive, spherical, bipolar neuron (rectangle) with two main axonal projections (right insert) giving rise to small numerous sub-branches. At different focal plane, another intense monopolar cell can be noticed superficial to the previously described neuron, with axonal projection toward the parietal-visceral connective (left insert). Note along the posterior lateral edge of the visceral ganglion situated dorsally a group of small separated intense neurons (small, solid white arrowheads) in addition to a large heavily stained neuron (arrow) with noticeable axon projecting toward the ganglionic body (large, solid white arrowhead). **C:** Ventral surface of the visceral ganglion (Vg). Histamine-like immunoreactive material is present in two clusters of 4–5 large neurons, including a group of cells (30–40 μm diameter, arrow) at the anterolateral margin of the ganglion near the right parietal-visceral connective (RPaVc) and a second medial group (25–30 μm diameter, arrowhead) near the left parietal-visceral connective (LPaVc). **D:** Higher magnification of medial histminergic cluster on the ventral surface of the visceral ganglion. Stout axons (arrows) project from these cells to the LPaVc. The fibers system connecting the left parietal and visceral ganglia together through the connective and connects the two ganglia with their follower ones (**A, B** contrasted black arrowheads). The z-stack spanned the thickness of the ganglion and both dorsal and ventral cells are shown in the projection. Calibration bar = 50 μm, all panels.

The left parietal ganglion consistently contained a single, intensely immunoreactive neuron, which was spindle-shaped (~25 μm diameter across the minor axis) and located toward the center of the dorsal surface (Fig 6A, solid white arrow). This cell projected a thick axon toward the underlying neuropil (Fig 6A, solid white arrowhead). A cluster of large, lightly stained cells (30–35 μm diameter) was located at the posterior dorsal edge of the left parietal ganglion (Fig 6A, asterisk). Another group of three small neurons was located near the origin of the left parietal-pleural connective (not shown but see summary diagram below). On the ventral surface, two pairs of lightly stained, large neurons were detected slightly lateral and posterior to the center of the ganglion (Fig 6A, brackets). In addition, a faint, monopolar cell was located medially on the ventral surface just anterior to the origin of the parietal-visceral connective (Fig 6A, rectangle). This latter cell projected an axon toward the visceral ganglion (Fig 6A, insert). Numerous axons including some prominent thick fibers were observed in the connective between the pleural ganglia and the parietal ganglia (Fig 6A, contrasted black arrow). This fiber system appeared to be continuous with a tract connecting the visceral and parietal ganglia (Fig 6A, contrasted black arrowhead). In contrast to the larger left parietal ganglion, the right parietal ganglion only contained one cluster of three neurons situated on the dorsal surface near the parietal nerves and a solitary neuron located on the ventral lateral edge (not shown but see summary diagram below).

A pair of large (30 μm), intensely immunoreactive neurons was reliably observed on the dorsal surface in anterior regions of the visceral ganglion. One of these adjacent cells possessed two main axonal projections (Fig 6B, right insert; solid white arrowheads) which gave rise to numerous small sub-branches while the other cell appeared to possess only a single axonal projection toward the parietal-visceral connective (Fig 6B, left insert). A group of small, loosely clustered neurons (10–15 μm) was situated dorsally along the posterior edge of the visceral ganglion (Fig 6B, small, solid white arrowheads), in addition to a large intensely stained neuron (Fig 6B, solid white arrow) with a single, anteriorly projecting axon (Fig 6B, large, solid white arrowhead).

Like other parts of the CNS, the pleural, parietal, and visceral ganglia contained numerous HA-Lir axons and a system of thick bundles of immunoreactive fibers ran through the connectives and neuropilar regions of all ganglia (Fig 6A and 6B, contrasted black arrowheads).

### Peripheral nervous system

**Fig 7:**
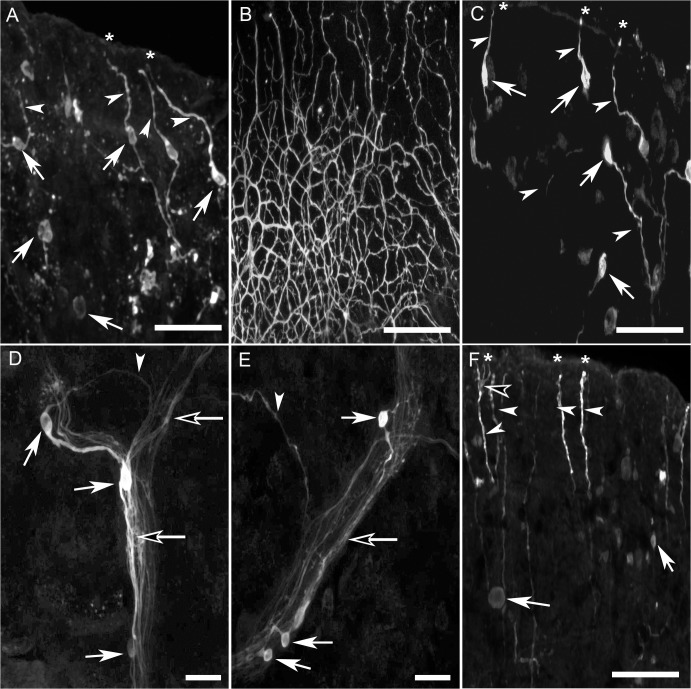
HA-like immunoreactive bipolar cells in the peripheral tissues of *B*. *alexandrina*. **A:** Numerous putative sensory cells (solid white arrows) in the dorsal surface of the lip, each cell possess both apical processes that penetrated the epithelium (solid white arrowhead) and projects from the apical surface by dendrites (asterisks) and also axons which appear to project centrally. **B:** Numerous immunoreactive axons forming a network lining the mouth. **C:** Bipolar cells with long axonal processes (solid white arrowheads) found along the length of the tentacle. Apical dendrites (asterisks) of the putative sensory cells penetrate the epithelium. **D:** Axons (solid white arrowheads) of immunoreactive cells join the adjacent nerves traversing the mantle (contrasted white arrows). **E:** Bipolar sensory cells (solid white arrows) with immunoreactive axons (solid white arrowheads) that join the main peripheral nerve of the foot (contrasted white arrows). **F:** Sensory cells in the tip of the foot (solid white arrows) with some cells devoted from the epithelial processes. Also, some long immunoreactive fibers can be demonstrated without any somata (solid white arrowheads), penetrating the epithelial and reaches the surface (asterisks). Some of these fibers branch near the surface (contrasted black arrowhead). Calibration bars: A-C, F = 50 μm; D, E = 25 μm.

Numerous bipolar cells (~10 μμm diameter) exhibited immunoreactivity in the tentacles, mantle, foot, lips, and around the mouth of *B*. *alexandrina*. These putative sensory cells projected what appeared to be apical dendrites to the body surface without discernible cilia (Fig 7A, 7C and 7F, asterisks) and possessed long axonal processes that joined branched peripheral nerves (Fig 7A–7F, solid white arrowheads). Their density was especially high in the lips and along the dorsal and medioventral surfaces of the tentacle tips (Fig 7A and 7C, solid white arrows). Numerous immunoreactive axons also formed a fine network beneath the epithelial layer lining of the mouth (Fig 7B). In both the mantle and foot, numerous histaminergic cells were located along nerves. The axons of bipolar cells of the mantle and foot (Fig 7D and 7E, solid white arrows) extended to join the peripheral nerves (Fig 7D and 7E, contrasted black arrows). In the foot, the muscular layer contained a number of immunoreactive axons (Fig 7F, solid white arrowheads) and what appeared to be unipolar cells (Fig 7F, solid white arrows) and axons. In addition, some HA-like immunoreactive neurites projected toward the apical surface penetrating the epithelium (Fig 7F, asterisks) but possessed no detectable soma in the region. Some of these fibers branched near the surface (Fig 7F, contrasted black arrowhead).

## Discussion

**Fig 8:**
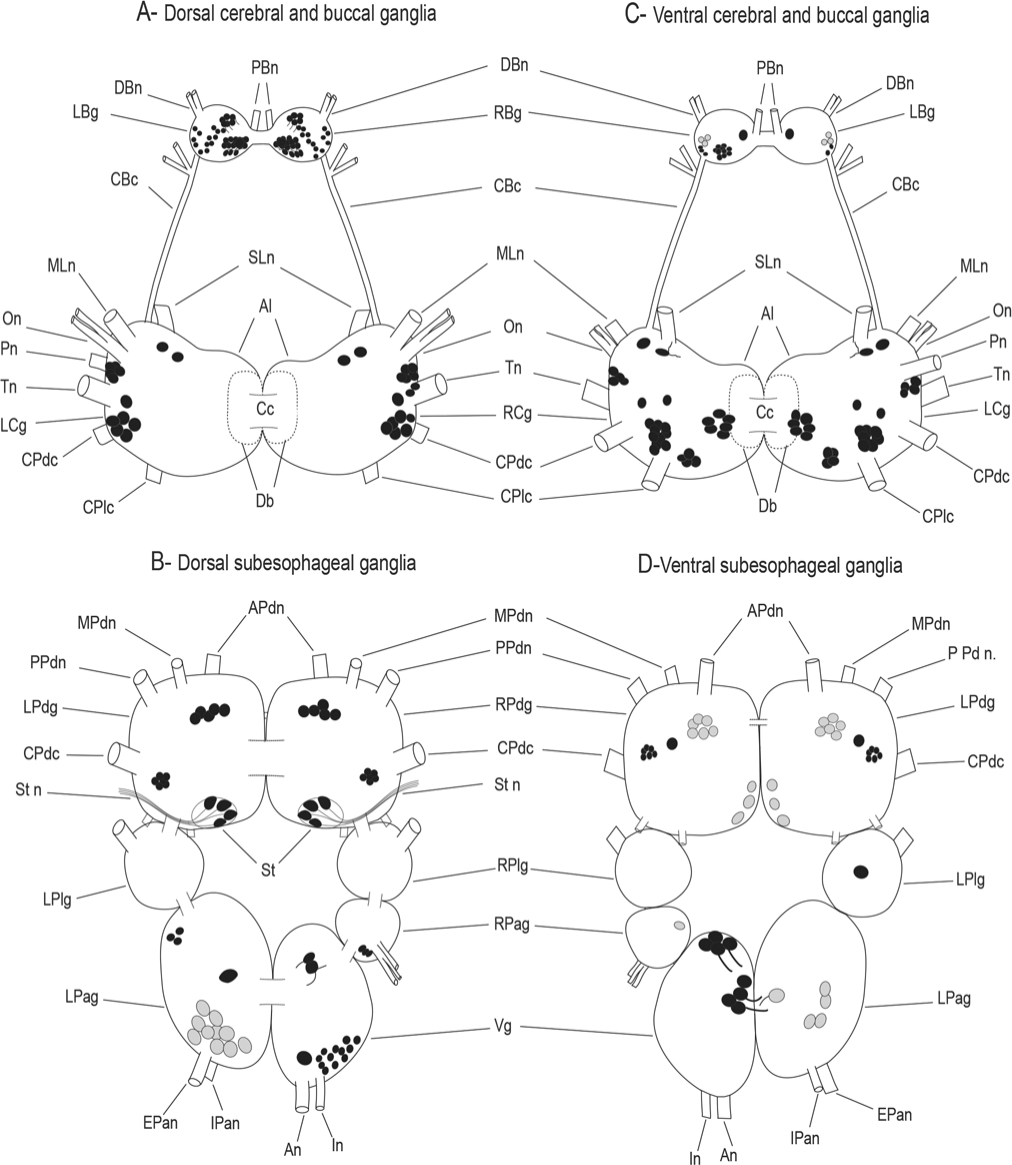
Schematic representation of HA-like immunoreactivity in the central nervous system of *Biomphalaria*. A and B represent dorsal view while C and D are for ventral view, **A and C:** The cerebral and buccal ganglia. **B and D:** The sub-esophageal ganglia. Gray profiles represent, dimly stained neurons, found consistently in most of the preparations. The cells are not drawn to scale. In the buccal ganglion, most of the neurons were located in clusters on the caudal surface. A few cells of different shapes and size are present in the two cerebral ganglia and distributed asymmetrically on either side of the ganglionic pair. Symmetric distribution of neurons can be noticed in the pedal ganglia. Two large oval circles at the caudal surface of the two pedal ganglia represent the statocysts. Note the presence of hair cells inside the statocysts, colored in white and black, projecting axons to the static nerve. The visceral and left parietal ganglia showed similar large intensely stained cell near the neuropil area, in addition to several other neurons. Abbreviations: An: anal nerve, APdn: anterior pedal nerve, Cc: cerebral commissure, CPdc: cerebral-pedal connective, DBn: dorsobuccal nerve, EPan: exterior parietal nerve, In: intestinal nerve, IPan: Interior parietal nerve, LBg: left buccal ganglion LCg: left cerebral ganglion, LPag: left parietal ganglion, LPdg: left pedal ganglion, LPlg: left pleural ganglion, MLn: medial lip nerve, MPdn: median pedal nerve, On: optic nerve, PBn: parabuccal nerve, Pn: penial nerve, PPdn: posterior pedal nerve, RBg: right buccal ganglion, RCg: right cerebral ganglion, RPag: right parietal ganglion, RPdg: right pedal ganglion, RPlg: right pleural ganglion, SLn: superior lip nerve, St: statocyst, Stn: static nerve, Tn: tentacular nerve, Vg: visceral ganglion.

The aim of the present study was to localize HA-Lir cells and processes in the nervous systems of *Biomphalaria alexandrina* and *Biomphalaria glabrata*. The results indicate that HA is widely distributed in the nervous system with immunolabeled somata abundant in the CNS (summarized in Fig 8) and peripheral tissues and intensely labeled fibers present in the ganglionic neuropils, connectives, commissures, and peripheral nerves.

### Biogenic amines in *Biomphalaria* and other snails

Biogenic amines are important neurotransmitters, hormones and modulators in gastropods. Detailed maps of their distributions have been described in several species for serotonin [42][43][44][45][46], catecholamines (i.e., dopamine and noradrenaline) [45][47][48]and HA [14,17]. An increasing literature is also emerging regarding the distribution of biogenic amines in *Biomphalaria*, stimulated by efforts toward identification of measures for controlling the snail and/or reducing its capacity to serve as the intermediate host of trematode larvae.

Early investigators used microfluorimetry and histochemical fluorescence microscopy to demonstrate the presence of serotonin in the cephalopedal tissues of *B*. *glabrata* [33]. More recently, Delgado et al. [34] used immunocytochemistry to reveal serotonergic neurons in the CNS and numerous fibers projecting to the periphery, thus implicating this amine in neural circuitry underlying locomotion, reproduction, and feeding in this snail. The numbers, positions and sizes of serotonergic cells in *B*. *glabrata* were generally similar to those described in other gastropods. The similarities between serotonergic neurons are especially striking within the pulmonates with the notable feature that bilateral asymmetries in sinistral snails such as *B*. *glabrata* and *Helisoma trivolvis* are mirror images of dextral snails such as *Lymnaea stagnalis* and most other pulmonates [23][21][22].

Catecholaminergic neurons were also previously localized in *B*. *glabrata* and *B*. *alexandrina* using a combination of immunohistochemistry for detection of tyrosine hydroxylase (the rate limiting enzyme in the synthesis of catecholamines) and aldehyde-induced histochemical staining [35]. The result of that study confirmed the conserved characteristics of aminergic neurons within gastropods, with catecholaminergic cells and cell clusters observed in the CNS of *Biomphalaria* having recognizable homologs in other gastropods. Again, asymmetric cells in sinistral basommatophoran species such as the large pedal neuron in *Biomphalaria* (LPeD1) [34], and the giant dopamine cell (GDC) of *Planorbis corneus* [20][49] and *H*. *trivolvis* [50][51] have mirror image homologues in the dextral species *L*. *stagnali*s [52][47]. In addition, all gastropods examined to date also possess numerous subepithelial sensory neurons that appear to contain catecholamines [47][48][53][54][35].

### Histamine-like immunoreactivity in the CNS of *Biomphalaria*

In the present study, we extend this research on biogenic amines by demonstrating that the ganglia of *B*. *alexandrina* and *B*. *glabrata* contain numerous HA-Lir cells and that their distributions are similar between the two species with homologous cells and cell clusters identified in the ganglia of both species. As with the staining for serotonin and catecholamines discussed above, the distribution of HA immunoreactivity in *Biomphalaria* shares many similarities with that previously described in *L*. *stagnalis*. Most of the histaminergic clusters identified in the buccal, cerebral, and pedal ganglia of *B*. *alexandrina*, were consistent with descriptions in *L*. *stagnalis*. For instance, in the buccal ganglia of *L*. *stagnalis*, the majority of the labeled nerve cells were located in clusters that appear to be similar to those identified in *Biomphalaria*. Such findings are consistent with a wider literature demonstrating the roles of HA in the generation of feeding behaviour in gastropods [55]. The results of our double labeling experiments demonstrate an anatomical substrate for the coordination of activity between the cerebral and buccal ganglia. In fact, the presence of abundant HA immunoreactive fibers in the neuropil and tracts running through all of the ganglia, commissures, and connectives is indicative of numerous roles for HA in intra- and interganglionic interactions, integration of afferent information, and modulation of peripheral targets [17]. In addition, the presence of clusters of neurons in the pedal ganglion and in particular the observation of a group of cells at the origin of the medial and inferior pedal nerves in *L*. *stagnalis*, with axons projecting toward the pedal nerves, are consistent with involvement of HA in the locomotory activity of these snails (see below). HA in the several neurons and neuropilar regions of the visceral-parietal ganglion complex suggests a role for this amine in regulating respiratory and circulatory systems controlled by these ganglia, e.g., heart activity as reported in other pulmonates [56][57][58]. Finally, the present study supports the function of HA as a primary neurotransmitter of the statocyst hair cells in gastropod molluscs. HA was previously reported in statocysts of several other gastropods [14][16][19][17]. We similarly found that hair cells and their axons in the static nerve contain HA in *Biomphalaria*. The sensory hair cells in the statocysts displayed variable HA reactivity with the majority of cells in the medial half having strong immunolabeling and the lateral cells exhibiting little or only weak labeling. Similar staining of subsets of hair cells have been previously reported in other species [16][59]. These findings are consistent with a conserved role for HA in gravireception within this taxon [19], and possibly more widely within the molluscs [40].

### Histamine immunoreactivity in the periphery

A key role of HA as a signaling molecule in the periphery of *Biomphalaria* was supported by the presence of dense HA-Lir innervation in the tentacles, mantle, foot, and around the mouth. A large number of labeled bipolar sensory cells, with putative sensory dendrites that penetrated the epithelium and axons joining branched peripheral nerves, was demonstrated in the body wall. Numerous immunoreactive fibers without obvious associated somata were also present, extending from the major nerves branching in the peripheral tissues. Similar histaminergic fibers were observed in *L*. *stagnalis* [18]. The histaminergic peripheral sensory cells probably project centrally, accounting for the high density of HA-like immunoreactivity in nerves to all three cephalic sensory organs (tentacles, lips, and foot) and their peripheral branches as previously noted in *Lymnaea* [17]. The dense population of HA-Lir elements in the head and foot regions suggests participation in both sensory (e.g., olfaction and taste) and possible efferent (neuromuscular and ciliary-motility) processes. Diverse regulatory roles for HA, including sensory, efferent, and integrative functions, are also suggested in the nervous system of other pulmonate species, such as *Helix pomatia* [17] and *L*. *stagnalis* [17][18].

### Possible roles for biogenic amines in *Biomphalaria-Schistosoma* interactions

As in molluscs, biogenic amines also appear to play many vital roles in platyhelminthes, with studies demonstrating particularly important functions in the control of motility and therefore survival within their hosts [60][32][30]. Serotonin and catecholamines have been extensively studied in platyhelminthes (for review, see [60]), with serotonin appearing to act as an excitatory neuromuscular transmitter in *S*. *mansoni* [61], while norepinephrine and dopamine appear to be inhibitory neurotransmitters causing a lengthening of the worm through muscular relaxation [61][62]. These transmitters therefore likely contribute to the movement of the parasite within its definitive host. Several studies have also demonstrated the involvement of serotonin and dopamine in the larval development of *S*. *mansoni*, specifically at the stage of transformation from the free-living miracidium to the parasitic mother sporocyst following penetration of the snail intermediate host. The emergence of daughter sporocysts from mother sporocysts is achieved via muscular contractions that could be promoted by uptake of serotonin and dopamine originating from neural elements that are present in the integument of the snail host [63][64][65][66][33]. While snails may provide a source of serotonin and catecholamines to *S*. *mansoni* larvae, the larvae could, in turn. also affect the snails through aminergic pathways as suggested by Delgado et al. [34] and Vallejo et al. [35]. For example, serotonin may be involved in parasitic castration [67], leading to a redirection of energy resources in infected snails [68][69][70][71][72]. Similarly, the presence of tyrosine hydroxylase-like immunoreactivity in the dorsal bodies of *Biomphalaria* [35] suggests that dopamine could also be involved in the diminished egg laying observed in infected snails [73].

Less is known about HA in platyhelminthes, in comparison to serotonin and catecholamines. HA is variably distributed among parasitic flatworms [74][75][76][77][78] but is also implicated in the control of muscle function and movement [31]. Some species are capable of endogenous biosynthesis of HA and have high tissue levels of the amine [79][80][81], whereas in other species, HA is present at low levels and may be entirely of host origin [82]. Numerous earlier studies, in experimental animals and in patients with acute or chronic schistosomiasis, have reported the release of HA during infections with *S*. *mansoni* [83][84][85] and the release of HA has also been demonstrated routinely in cell cultures within 1 h after incubation with antigens from adult, egg, or cercarial stages of *S*. *mansoni* [86]. Thus there appear to be ample sources for HA in the definitive host.

The present study identifies several potential neural sources for HA in the intermediate host. Specifically, the presence of central and peripheral histaminergic neurons in *B*. *alexandrina* could serve as exogenous sources of free HA in innervated tissues or fluid spaces, as has been suggested for other biogenic amines found in *B*. *glabrata* [67][87][34][35]. If the parasites can then in turn influence overall levels of HA in the snail host, as has been suggested for other amines, our results indicate this might particularly affect feeding and locomotion in *Biomphalaria* since the neural circuits underlying these behaviours are abundant in HA.

In conclusion, HA is an important neuroactive substance across the animal kingdom, and like other biogenic amines, HA is abundant in nervous system and periphery of *Biomphalaria*. Our detailed descriptions of its distribution in this species serve as a basis for future studies into its involvement in the neural circuity mediating various behaviors and for comparisons with other species. Finally, our findings also provide an initial step in identifying histaminergic targets for potential control strategies involving this host of *S*. *mansoni* and for a better understanding of parasite-induced modifications to host behavior.
